# Managing behavioural and developmental paediatric conditions in rural outpatient clinics: An insight to the challenge ahead

**DOI:** 10.1111/jpc.15923

**Published:** 2022-02-25

**Authors:** Luke David Kardell, Joanna Lee, Janani Pinidiyapathirage, Kay Brumpton

**Affiliations:** ^1^ School of Medicine and Dentistry Griffith University Southport Queensland Australia; ^2^ Rural Medical Education Australia Toowoomba Queensland Australia

**Keywords:** aboriginal and Torres Strait Islander children, audit, children, general paediatrics, neurodevelopmental disorders, rural, rural health

## Abstract

**Aim:**

The aim of this study is to characterise the nature and caseload of general paediatric outpatient clinics in rural Queensland and to compare the findings with national data.

**Methods:**

A retrospective clinical audit of medical records in paediatric clinics at two rural hospitals was undertaken over a 6‐month period. Data extracted included demographics, diagnostic category and onward referral outcomes. The same diagnostic categories were used as the national Children Attending Paediatricians Study (CAPS) to facilitate comparison.

**Results:**

A total of 346 records were reviewed, 56 (16.2%) documented Aboriginal and Torres Strait Islander descent. Compared to national data, significantly more children with behavioural/developmental diagnoses were seen in the rural clinics (CAPS 33.8%; rural 59.2%; *P* < 0.001). In contrast, children presenting with medical conditions (CAPS 48.2%; rural 28.6%; *P* < 0.001) and mixed medical/developmental conditions (CAPS 17.9%; rural 12.1%; *P* = 0.006) were seen less frequently in the rural clinics. Referral rates from the rural sites were lower than the rates reported by CAPS for children with behavioural/developmental diagnoses (CAPS 35.9%, rural 24.9%; *P* = 0.002) and mixed diagnoses (CAPS 40.6%, rural 19.0%; *P* = 0.005), while there was no difference in referral rates for children with medical diagnoses (CAPS 16.1%, rural 18.2%; *P* = 0.575).

**Conclusions:**

Rural paediatricians' caseloads are dominated by developmental/behavioural conditions, however children with these conditions are less likely to be referred to allied health and psychology services. The reasons for lower referral rates and specific pressures upon rural health services need to be investigated in future studies to provide better health services for rural children.

## What is already known on this topic

1. Australian paediatricians have been seeing more children with behavioural and developmental conditions over recent years.
2. It is unclear whether the caseload of rural paediatricians matches national trends.

## What this paper adds

1. In comparison to national data, a significantly higher proportion of children with behavioural and developmental conditions and Aboriginal and Torres Strait Islander background are seen in paediatric clinics in rural settings.
2. Referral rates to allied health and subspecialities are lower for rural children with behavioural and developmental diagnoses, particularly to psychological services.

The proportion of Australian children presenting to paediatric services with developmental, behavioural and psychosocial issues has been on an upward trajectory, a trend which is reflected globally.^1^ This shifting nature in paediatric presentations has been reported in Australia since 1988, with a marked increase in children with school problems, behavioural problems and hyperactivity.^2^ In 1999, a 12‐month survey of paediatricians in the Barwon region of Victoria found that 34.8% of consultations were for behavioural problems, and the most frequently made diagnosis was attention‐deficit hyperactivity disorder (ADHD; 30.0%), followed by disability and/or epilepsy (14.1%) and asthma (12.5%).^3^ These trends were confirmed again in the 2008 nationwide audit by the Australian Paediatric Research Network (APRN) in The Children Attending Paediatricians Study (CAPS).^4^ In this study, paediatricians in both public and privates sectors across all states and territories of Australia were asked to record either 100 consecutive consultations or all consultations over a 2‐week period.^4^ The results were dominated by consultations for ADHD (18.3%) and learning difficulties (7.5%). On subsequent analysis, one‐third of consultations were for developmental/behavioural diagnoses compared to nearly half for medical diagnoses, while 18% had mixed diagnoses.^5^ The APRN further illustrated that presentations relating to developmental/behavioural issues rose from 2008 to 2013, with an increasing proportion of consultations for ADHD, autism spectrum disorders, anxiety, sleep problems and intellectual disabilities.^6^

The increasing share of children with developmental and behavioural problems seen in general paediatric outpatients has broad implications for future health‐care workforce provision, training and funding specially to provide additional training in community and child health for paediatricians in the field of developmental and behavioural paediatrics. With an evolution of training requirements over the past 30 years, general paediatricians with additional training can be considered as the most suitable health workforce to manage developmental and behavioural conditions.^7^ The current model of care in Queensland places the local general practitioner (GP)/family doctor at the forefront of providing continued, comprehensive care for children.^8^ Working closely with child health nurses, GPs have the opportunity to identify and refer children with developmental and behavioural problems to specialised child health services when they routinely perform health checks at 6 months, 12 months, 18 months, 2½–3½ years and 4–5 years. In addition, school‐aged children identified as having academic or behavioural problems can be directly referred to specialist services by school guidance officers.^9^ Despite having an organised structure, children with both behavioural and developmental conditions have been rated among the most difficult to manage due to the multifactorial nature and the complexity of social interventions required to manage them.^10^ Developmental and behavioural presentations also tend to have multiple comorbidities with significant burden of illness,^11^ necessitating longer appointments and frequent referrals to allied health.^5^

An important area that the previous studies do not report on is the caseload of rural paediatric outpatient services. Children in regional and rural communities (defined as areas outside of major cities) have poorer health and developmental outcomes compared to their metropolitan peers, with significant psychosocial vulnerabilities and limited access to appropriate health services in close proximity.^12^ Paediatricians have reported that they are often the only clinicians to manage complex behavioural disorders which leads to long wait‐lists leading to delays in children receiving diagnoses and subsequent management.^12^ Therefore, reforms are required to improve access to medical and allied health care for children in regional and rural areas that must be guided by evidence which is currently lacking. The aims for this exploratory clinical audit of two rural general paediatric outpatient clinics were to characterise the caseload seen over a 6‐month period and to compare the results to previously published national CAPS data.^4^, ^6^

## Methods

### Study design

We conducted a retrospective clinical audit of the medical records of the general paediatric outpatient clinics at Kingaroy and Warwick hospitals (Modified Monash Model‐4) in Southern Queensland during the period 1 January to 30 June, 2019. This project was approved by the Darling Downs Health Human Research Ethics Committee (LNR/2019/QTDD/51839).

### Study population and sites

The study population consisted of all children aged 0–19 years seen in the outpatient clinics during the specified period at the two rural hospitals. Both hospitals are in the Darling Downs Region with each servicing a population around 10 000–12 000.^13^ The region has a higher proportion of children aged 0–19 compared to the rest of the country (Kingaroy 28.1%, Warwick 27.3%, Australia 24.8%),^13^ as well as a higher proportion of Aboriginal and Torres Strait Islander children (Kingaroy 10.9%, Warwick 13.8, Australia 5.9%).^13^ The study areas report a high level of socio‐economic disadvantage (Socio‐Economic Indexes for Areas percentile 14th–17th).^14^ The rural hospitals had differing access to paediatric services, with the clinic at the Kingaroy Hospital serviced monthly by one to two visiting general paediatricians and one local GP with advanced specialised training in paediatrics, while the clinic in Warwick Hospital had weekly outpatient service with one to two visiting paediatricians. Both clinics service a wider region than the towns themselves, including patients from the surrounding rural areas and smaller, more remote towns. Neither hospital has a dedicated paediatric inpatient service, paediatric mental health services or paediatric allied health practitioners. Most of the allied health practitioners in the region are generalists treating patients of all ages. Any child whose needs could not be met by the visiting paediatricians are referred for further care to Toowoomba Hospital where specialist paediatric services are available (<2 h from each location) or to the Queensland Children's Hospital in the state's capital city Brisbane (2–3 h distance) to obtain more advanced paediatric support services.

### Data collection

A list was first generated from the clinic booking system; if the patient was seen multiple times over the 6‐month period, clinical data were collected for their most recent consultation.

Data included children's demographics, diagnoses recorded during the consultations and patient dispositions. Data were extracted by two medical students with appropriate training in handling medical records (LDK and JL).

Diagnoses extracted from medical records were subsequently re‐categorised by the same student investigators to follow the codes used in the CAPS.^4^ Patient encounters were classified as behavioural or developmental if all diagnoses were behavioural or developmental, medical if all diagnoses were medical, and mixed if there was at least one behavioural or developmental diagnosis and one medical diagnosis.

### Data analysis

Proportions were used to describe categorical data. Means and standard deviations or medians and interquartile ranges (IQR) were used to describe continuous data which were normally distributed and skewed, respectively. Chi‐square tests were used to determine associations between categorical variables, and analysis of Variance and Kruskal‐Wallis tests were used to compare continuous data for normally distributed and skewed data, respectively. To estimate the difference between our results and national CAPS data, we used the standard error of difference between two proportions tests. Comparisons were made with the 2008 CAPS audit^4^ data and where possible, with 2013 CAPS audit data.^6^ The 2013 audit presented data on the changing proportion of consultations for behavioural and developmental diagnoses and did not report any data on referral rates. All data analyses were completed using IBM SPSS version 26 (IBM SPSS Statistics for Windows, IBM Corp, USA).

## Results

Over the 6‐month study period, a total of 346 children were seen in the selected rural paediatric outpatient clinics. A summary of patient demographics according to their diagnostic category is given in Table 1. In total, 205 (59.2%) children presented with behavioural or developmental issues, 99 (28.6%) presented with medical issues and 42 (12.1%) presented with both behavioural/developmental and medical issues. Children with developmental and behavioural issues were significantly older than those with medical issues (median age in years 9.0 (5.8–12.0) vs. 4.0 (1.4–10.5); *P* < 0.001) and were more likely to be male (145 (70.7%) vs. 47 (47.5%); *P* < 0.001). Aboriginal and Torres Strait Islander children made up a large proportion of those presenting with medical issues compared to children presenting with developmental and behavioural issues; however, the observed difference was not statistically significant (*P* = 0.064). Out of the top 20 most frequent diagnoses, 13 were behavioural/developmental conditions, including eight out of the top 10 diagnoses. The top five behavioural and developmental diagnoses were ADHD (25.7%), autism spectrum disorder (23.7%), behaviour problems (13.3%), language delay (9.5%) and anxiety disorders (7.5%); these were the top five most common diagnoses overall. The top five medical diagnoses were neurological conditions (excluding seizure disorders; 5.5%), seizures/epilepsy (4.6%), gastrointestinal disorders (2.9%), asthma (2.6%) and constipation (2.3%). Among Aboriginal and Torres Strait Islander children, the top 10 diagnoses were ADHD (23.2%), autism spectrum disorder (16.1%), neurological conditions (16.1%), mental health (10.7%), language delay and learning difficulty (both 8.9%), and behaviour problems, intellectual disability, gastrointestinal and dermatological disorders (each 7.1%).

**Table 1:** The association between patient demographics and clinical diagnoses

	Behavioural or developmental	Medical	Mixed	Total	*P* value
Affected children, *n* (%)	205 (59.2)	99 (28.6)	42 (12.1)	346	
Median age (years), (interquartile range)^†^	9.0 (5.8–12.0)	4.0 (1.4–10.5)	9.1 (5.3–12.2)	8.3 (4.5–11.6)	<0.001
Males, *n* (%)^‡^	145 (70.7)	47 (47.5)	30 (71.4)	222 (64.2)	<0.001
Aboriginal and Torres Strait Islander children affected, *n* (%)^‡^,^§^	26 (12.7)	23 (23.2)	7 (16.7)	56 (16.2)	0.064
Average number of diagnoses (SD)^¶^	1.6 (0.8)	1.3 (0.6)	2.9 (0.9)	1.7 (0.9)	<0.001

† Kruskal‐Wallis test.

‡ Chi‐square test.

§ Not stated 31 (9.0%).

¶ Analysis of variance.

SD, standard deviation.

Compared to 2008 CAPS data,^4^ behavioural and developmental conditions were seen more frequently at both rural paediatrics clinics studied (CAPS 2008 33.8%; rural 59.6%; *P* < 0.001). In the 2013 CAPS audit, developmental and behavioural problems had risen to 41% (this is considering only the top 10 new diagnoses).^6^ Even though sufficient information was unavailable to conduct statistical tests, the numbers observed support a rapidly increasing trend in developmental and behavioural problems among children (Table 2). Furthermore, patients presenting with medical conditions (CAPS 2008 48.2%; rural 28.3%; *P* < 0.001) and with mixed medical/developmental conditions (CAPS 2008 17.9%; rural 12.1%; *P* = 0.006) were seen less frequently in these rural sites compared to the 2008 national CAPS data.^4^

**Table 2:** Diagnosis categories of paediatric referrals to selected paediatric clinics compared to national audit data

Diagnoses	% CAPS 2008	% CAPS 2013^†^	% Rural^‡^
Behavioural or developmental	33.8	41	59.2
Medical	48.2	12	28.6
Mixed	17.9	Not available	12.1

† Only the proportion of cases in the top 10 new diagnoses are included.

‡ Modified Monash Model‐4.

CAPS, Children Attending Paediatricians Study.

Table 3 presents the outcome of clinic appointments. When comparing outcomes by diagnosis, significantly more patients with medical diagnoses were discharged (17.2%), compared to either developmental (2.4%) or mixed (2.4%, *P* < 0.001 for both diagnoses). There were no significant differences in rates of onward referral to allied health, subspecialist paediatrician or psychologist/mental health between the three diagnostic categories. Comparing referral rates of the rural sites to the national data found that patients with behavioural or developmental diagnoses were referred significantly less often from rural sites (CAPS 2008 35.9%, rural 24.9%; *P* = 0.002). Patients with medical diagnoses had no significant differences in referral rates (CAPS 2008 16.1%, rural 18.2%; *P* = 0.575), while children with mixed diagnoses were overall referred significantly less from rural sites (CAPS 2008 40.6%, rural 19.0%; *P* = 0.005).

**Table 3:** A comparison of referral patterns of rural paediatric clinics to 2008 national audit data

Referrals	Behavioural or developmental (%)			Medical (%)			Mixed (%)			Total (%)		
	Rural (*n* = 205)	CAPS (*n* = 2783)	*P* value^†^	Rural (*n* = 99)	CAPS (*n* = 3975)	*P* value^†^	Rural (*n* = 42)	CAPS (*n* = 1477)	*P* value^†^	Rural (*n* = 346)	CAPS (*n* = 8235)	*P* value^†^
Referred to another service	24.9	35.9	**0**.**002**	18.2	16.1	0.575	19.0	40.6	**0**.**005**	22.5	27.2	0.054
Allied health	11.2	18.3	**0.010**	7.1	5.7	0.554	11.9	20.3	0.180	10.1	12.6	0.168
Subspecialist	7.8	4.8	0.057	11.1	9.2	0.519	4.8	14.8	0.070	8.4	8.7	0.846
Psychologist/mental health	5.9	16.0	**<0.001**	0	0.3	0.585	2.4	9.3	0.126	3.8	7.2	**0.016**

† Standard error of difference between two proportions test; significant results are highlighted in bold.

CAPS, Children Attending Paediatricians Study (data from 2008 study).

## Discussion

In this clinical audit, we have been able to highlight the workload on paediatric services due to the large proportion of children presenting with developmental and behavioural problems, with approximately two of three children presenting with such conditions. Within the top 10 diagnoses, eight were related to developmental and behavioural conditions and two for medical conditions (both were neurological conditions). Rural sites saw a significantly higher proportion of children with developmental and behavioural diagnoses and these children were referred less often compared to the available national CAPS data.

The trend of increasing developmental and behavioural conditions seen in the Barwon region study^3^ and both CAPS audits^4^, ^6^ was reaffirmed in this audit. In the 2013 CAPS audit, there was a higher proportion of consultations for behavioural and developmental conditions compared to 2008 CAPS audit, thus the major difference seen in these results may reflect the progressive trend seen nationally. Nonetheless, children living in rural and remote areas are more likely than their metropolitan counterparts to be developmentally vulnerable in multiple domains.^15^ Data from the Australian Early Development Census supports some of our findings with the proportion of children developmentally vulnerable on one or more domains in Warwick area being much higher than the reported national averages.^16^ If at a local population level there is an increased prevalence of these conditions, then it should be expected that paediatricians would encounter children with these conditions more frequently.

The proportion of Aboriginal and Torres Strait Islander children seen in this audit was much higher than in the 2008 CAPS audit (16.2% vs. 2.9%).^4^ Both towns studied have larger proportions of Aboriginal and Torres Strait Islander Australians compared to the wider Australian population (Australia 5.9%, rural 12.3%),^13^ however, the proportion seen in the clinics was in excess of what would be expected based on population data alone. These data are encouraging in that, vulnerable children are gaining access to paediatric services, however, the data more likely reflect the history of this region with First Nations people being forcibly removed from their homelands across eastern Australia to form a government reserve in the area in early 20th century.^17^

In this study, the most frequently occurring diagnosis category was developmental and behavioural disorders both in Aboriginal and Torres Strait Islander children and in non‐Indigenous children. The 2008 CAPS study found the most frequently occurring diagnosis to be ADHD (16.9%), followed by otitis media (14.4%) and baby checks (9.9%).^4^ Our results differ in part due to the children seen being older (median age 7.4 years, IQR 3.8–12.6) and possibly due to baby checks and otitis media now being managed mostly by GPs. Other factors may include delays associated with seeking health care, outpatient waiting periods, access to early intervention and early childhood education, higher levels of non‐school attendance, health literacy levels, referral inertia by practitioners and other more complex socio‐economic factors.^18^

The referral rates to psychology and other allied health services for behavioural and developmental diagnoses in our study were significantly lower than in the national CAPS study. The lower referral rates in our study may have been due to lower acuity cases not requiring referral, children already receiving allied health treatment or challenges accessing these services. However, exploring these factors was beyond the scope of this study but would certainly warrant future investigation. Standard practise at the rural hospitals is for children to be referred from their GP direct to the paediatric outpatient clinic with no pre‐assessment by allied health services. Furthermore, due to the generalist nature of most of the allied health practitioners in the region, they may not have confidence or experience in treating children with special needs. It should also be noted that allied health visits for these children tend to be more frequent and require greater time commitment which makes it harder to travel out of town to receive therapy. Therefore, it is unlikely that children were already receiving adequate allied health input. Moreover, the socio‐economic context of the region suggests these children have higher acuity needs and vulnerabilities.

Interestingly, the referral rates to subspecialists were higher for children with behavioural or developmental diagnosis but significantly lower for patients with mixed diagnoses. For children with medical diagnoses, there were no significant differences between onward referral between the rural clinics and CAPS data. While one would expect more referrals for rural children with mixed diagnoses to specialist centres, the competing demands for services may be a limiting factor for referrals. Furthermore, these differences may demonstrate the complex needs of children with behavioural and development disorders and the need for multidisciplinary services that may not be available in the rural communities. In addition, visiting practitioners working in unfamiliar environments may be more likely to send families to the larger secondary and tertiary referral centres where supportive services are accessible to manage behavioural and developmental conditions.

While all patients in this study were seen in person, telemedicine has long been considered as a way to reduce the distance between rural communities and health services.^19^ Of the three patients who accessed telemedicine, all had developmental diagnoses; two were referred for telepsychology, and the other to a tertiary telehealth sleep clinic. It appears that there is room for growth in providing outpatient care for rural children, and it will be interesting to see whether the changes that have occurred in the COVID era, such as improved access to virtual treatment platforms, will generate improved access to paediatricians and allied health services for rural children.

This clinical audit has several strengths as well as some limitations. It was able to successfully characterise all patients seen during a 6‐month period at each clinic. This period could capture seasonal variations that influence disease patterns. We have used the same diagnostic codes as the 2008 CAPS audit, allowing comparison between the datasets and providing an insight into the state of rural paediatric clinics. Data from 2013 CAPS audit did not provide same information as 2008 audit hence, we were unable to make similar comparisons except for a limited number of indictors. Both medical students involved in data extraction reviewed each other's work and agreed upon the final diagnoses to limit any categorisation errors. However, this relied upon the accuracy of diagnoses already written in the medical records.

The results of this audit need to be interpreted in light of its limitations. Since only two clinics were sampled, our results may not be generalisable to all rural clinics, where differences in local policy, paediatrician training and experience, access to allied health services, local psychosocial characteristics of health and availability of disability funding schemes^20^ may differ. The clinics service a wider region than the towns themselves, and thus, patients may have come from smaller, more remote towns. Finally, this study could not describe specific pressures upon rural paediatricians (including adequate training, support, remuneration etc.), which are important to consider for future resource allocations.

## Conclusion

The caseload of the rural paediatric outpatient clinics reviewed in this clinical audit was dominated by developmental and behavioural conditions. The affected children had lower than expected onward referral rates particularly to allied health and psychological services. The results have important implications for future rural paediatric health‐care delivery, training and funding. This study identifies a significant gap in service delivery for rural paediatric patients in accessing multidisciplinary teams to address behavioural and developmental conditions. Future studies need to confirm these results in other rural locations and conduct focused studies to identify specific pressures upon rural health services in managing children with behavioural and developmental conditions.
